# LpxK Is Essential for Growth of *Acinetobacter baumannii* ATCC 19606: Relationship to Toxic Accumulation of Lipid A Pathway Intermediates

**DOI:** 10.1128/mSphere.00199-17

**Published:** 2017-07-26

**Authors:** Jun-Rong Wei, Daryl L. Richie, Mina Mostafavi, Louis E. Metzger, Christopher M. Rath, William S. Sawyer, Kenneth T. Takeoka, Charles R. Dean

**Affiliations:** Novartis Institutes for BioMedical Research, Emeryville, California, USA; University of Rochester

**Keywords:** *Acinetobacter baumannii*, LpxK, lipid A, outer membrane

## Abstract

*Acinetobacter baumannii* ATCC 19606 can grow without lipid A, the major component of lipooligosaccharide. However, we previously reported that depletion of LpxH (the fourth enzyme in the lipid A biosynthetic pathway) prevented growth of this strain due to toxic accumulation of lipid A pathway intermediates. Here, we explored whether a similar phenomenon occurred with depletion of LpxK, a kinase that phosphorylates disaccharide 1-monophosphate (DSMP) at the 4′ position to yield lipid IV_A_. An *A. baumannii* ATCC 19606 derivative with LpxK expression under the control of an isopropyl β-d-1-thiogalactopyranoside (IPTG)-regulated expression system failed to grow without induction, indicating that LpxK is essential for growth. Light and electron microscopy of LpxK-depleted cells revealed morphological changes relating to the cell envelope, consistent with toxic accumulation of lipid A pathway intermediates disrupting cell membranes. Using liquid chromatography-mass spectrometry (LCMS), cellular accumulation of the detergent-like pathway intermediates DSMP and lipid X was shown. Toxic accumulation was further supported by restoration of growth upon chemical inhibition of LpxC (upstream of LpxK and the first committed step of lipid A biosynthesis) using CHIR-090. Inhibitors of fatty acid synthesis also abrogated the requirement for LpxK expression. Growth rescue with these inhibitors was possible on Mueller-Hinton agar but not on MacConkey agar. The latter contains outer membrane-impermeable bile salts, suggesting that despite growth restoration, the cell membrane permeability barrier was not restored. Therefore, LpxK is essential for growth of *A. baumannii*, since loss of LpxK causes accumulation of detergent-like pathway intermediates that inhibit cell growth.

**IMPORTANCE** *Acinetobacter baumannii* is a Gram-negative pathogen for which new therapies are needed. The lipid A biosynthetic pathway has several potential enzyme targets for the development of anti-Gram-negative agents (e.g., LpxC). However, *A. baumannii* ATCC 19606 can grow in the absence of LpxC and, correspondingly, of lipid A. In contrast, we show that cellular depletion of LpxK, a kinase occurring later in the pathway, inhibits growth. Growth inhibition results from toxic accumulation of lipid A pathway intermediates, since chemical inhibition of LpxC or fatty acid biosynthesis rescues cell growth upon loss of LpxK. Overall, this suggests that targets such as LpxK can be essential for growth even in those Gram-negative bacteria that do not require lipid A biosynthesis *per se*. This strain provides an elegant tool to derive a better understanding of the steps in a pathway that is the focus of intense interest for the development of novel antibacterials.

## Abstract (precis)

*Acinetobacter baumannii* is a Gram-negative pathogen for which new therapies are needed. The lipid A biosynthetic pathway has several potential enzyme targets for the development of anti-Gram-negative agents (e.g., LpxC). However, *A. baumannii* ATCC 19606 can grow in the absence of LpxC and, correspondingly, of lipid A. In contrast, we show that cellular depletion of LpxK, a kinase occurring later in the pathway, inhibits growth. Growth inhibition results from toxic accumulation of lipid A pathway intermediates, since chemical inhibition of LpxC or fatty acid biosynthesis rescues cell growth upon loss of LpxK. Overall, this suggests that targets such as LpxK can be essential for growth even in those Gram-negative bacteria that do not require lipid A biosynthesis *per se*. This strain provides an elegant tool to derive a better understanding of the steps in a pathway that is the focus of intense interest for the development of novel antibacterials.

## INTRODUCTION

*Acinetobacter baumannii* is an important bacterial pathogen of increasing concern in hospital settings due to multidrug resistance (1, 2). Carbapenem-resistant *A. baumannii* has recently been defined as a priority 1 pathogen in the newly released WHO priority pathogen list for research and development of new antibiotics (http://www.who.int/mediacentre/news/releases/2017/bacteria-antibiotics-needed/en/). *A. baumannii* can develop resistance to antibiotics via mechanisms such as loss of porins, upregulation of efflux pumps, acquisition of β-lactamases and aminoglycoside-modifying enzymes, and mutational alteration of target proteins (3–5). An important factor contributing to the intrinsic resistance of Gram-negative bacteria such as *A. baumannii* is the outer membrane (OM), located externally to the periplasm and presenting a significant permeability barrier to toxic molecules. Lipopolysaccharide (LPS) is the major component of the outer leaflet of the OM, and its biosynthesis and insertion into a functional OM are essential for growth of most Gram-negative bacteria (6). LPS has a general structure comprised of relatively conserved lipid A, which anchors LPS in the OM outer leaflet, to which are attached a core oligosaccharide and highly variable O-antigen polysaccharides extending outward from the cell surface. Some Gram-negative bacteria, such as *A. baumannii*, appear to lack a dedicated O-antigen ligase and do not attach an O-antigen to the lipid A core, thus producing lipooligosaccharide (LOS) (7). OM biogenesis and, in particular, lipid A biosynthesis and LPS/LOS transport are considered attractive targets for antibacterial drug discovery (6, 8–10). This goal can be approached by identifying compounds with direct antibacterial activity that target essential enzymes in the lipid A pathway or by combination approaches aimed at disrupting the permeability barrier to potentiate the activity of other antibacterial compounds (8). Interestingly, it was recently discovered that lipid A is not essential for growth in *A. baumannii*, based on the *in vitro* isolation of colistin-resistant mutants that lacked LOS, which is required for colistin entry into cells and antibacterial activity (11). The lack of LOS was caused by loss-of-function mutations in the *lpxA*, *lpxC*, and *lpxD* genes that encode enzymes acting early in the lipid A biosynthetic pathway (11). In *Escherichia coli*, where this metabolic pathway is best understood, lipid A biosynthesis is initiated by enzymes LpxA, LpxC, and LpxD, which together catalyze the addition of two β-hydroxyacyl chains (of lengths that differ depending on the species and active-site hydrocarbon rulers) to UDP-GlcNAc to form UDP-2,3-diacyl-GlcN. The LpxA and LpxD substrate β-hydroxyacyl-ACP is generated through the bacterial type II fatty acid synthesis (FASII) pathway and is a common precursor linking LPS and phospholipid (PL) biosynthesis (12, 13). The LpxD product, UDP-2,3-diacyl-GlcN, is subsequently hydrolyzed by the LpxH membrane-associated phosphodiesterase to generate lipid X, followed by the LpxB-catalyzed condensation of this product with UDP-2,3-diacyl-GlcN to form a tetra-acylated disaccharide 1-monophosphate (DSMP) (14, 15). DSMP is then phosphorylated at the 4′ position by the integral membrane kinase LpxK (16), yielding lipid IV_A_. To form mature LPS, inner core sugars and secondary acyl chains are added to lipid IV_A_ to generate core-lipid A. Core-lipid A is subsequently flipped from the cytoplasmic face to the periplasmic face of the inner membrane by MsbA (17, 18) and subsequently decorated with O-antigen polysaccharide polymers and transported across the periplasm to the outer leaflet of the OM through the action of the ATP-dependent LptA-G system (6, 19, 20). The minimal structure needed for viability of *E. coli* is that represented by lipid IV_A_ (21); however, this differs across Gram-negative species. For example, phosphorylation (by WaaP) of the inner core oligosaccharide is required for growth in *Pseudomonas aeruginosa* (22–24), and some *A. baumannii* strains can grow in the absence of lipid A. It is not fully understood why some *A. baumannii* strains (e.g., ATCC 19606) are able to compensate for the complete loss of lipid A, but recent work demonstrated that the presence of a gene encoding penicillin binding protein 1A (PBP1A) defines *A. baumannii* strains that cannot tolerate loss of lipid A (25). Increased cell surface expression and decoration of lipoproteins occur when PBP1A is deleted from cells, and this was suggested to play a role in tolerating lipid A depletion.

Although lipid A itself and, correspondingly, the LpxA and LpxC enzymes are not essential for growth of *A. baumannii* ATCC 19606, these may still represent viable antibacterial targets. Since the OM is also important for protection against host immune factors, it is unlikely that cells lacking the LOS-containing OM would survive during many infections (10). Furthermore, such LPS-deficient organisms are hypersusceptible to multiple antibiotics; thus, inhibitors of lipid A biosynthesis may be expected to potentiate the activity of many antibiotics, opening the possibility of combination therapies. Although enzymes such as LpxA and LpxC are not essential in *A. baumannii* ATCC 19606, at least under standard laboratory conditions, other enzymes in the lipid A biosynthetic pathway may be essential for growth since several pathway intermediates, such as lipid X and UDP-2,3-diacyl-GlcN (which have critical micelle concentrations below 25 µM), are detergent-like (26) and blocking certain pathways steps might cause the toxic accumulation of these intermediates. Indeed, we previously showed that the phosphodiesterase LpxH is essential for growth of *A. baumannii* ATCC 19606 using an isopropyl β-d-1-thiogalactopyranoside (IPTG)-regulated expression system (27). Depletion of LpxH also caused cell envelope defects and accumulation of detergent-like lipid A intermediates (27). Furthermore, the growth defect due to LpxH depletion was ameliorated by chemical inhibition of LpxC, upstream of LpxH, presumably by blocking the synthesis of detergent-like intermediates and thereby preventing their accumulation (27).

Quantitative modeling of lipid A biosynthesis in *E. coli*, where LPS biosynthesis is essential, predicted that LpxK, a kinase acting downstream of LpxH, may be the rate-limiting enzyme and as such a particularly attractive drug target within the lipid A pathway (12, 13). We therefore asked whether loss (and by extension, chemical inhibition) of LpxK might also have the potential to be directly antibacterial. An IPTG-inducible LpxK expression strain of *A. baumannii* ATCC 19606 was unable to grow in cation-adjusted Mueller-Hinton (MH) medium lacking IPTG, indicating that LpxK is essential for growth. Under LpxK depletion conditions, we observed changes in cell morphology and significant accumulation of the detergent-like lipid A intermediates DSMP and lipid X, as measured by liquid chromatography-mass spectrometry (LCMS). Consistent with this, chemical inhibition of LpxC allowed growth in the absence of IPTG and reversed the accumulation of these intermediates. Intriguingly, we also found that inhibitors of fatty acid biosynthesis could similarly restore growth and prevent the accumulation of toxic intermediates.

## RESULTS

### LpxK is required for *A. baumannii* ATCC 19606 growth under standard laboratory conditions.

**FIG 1:**
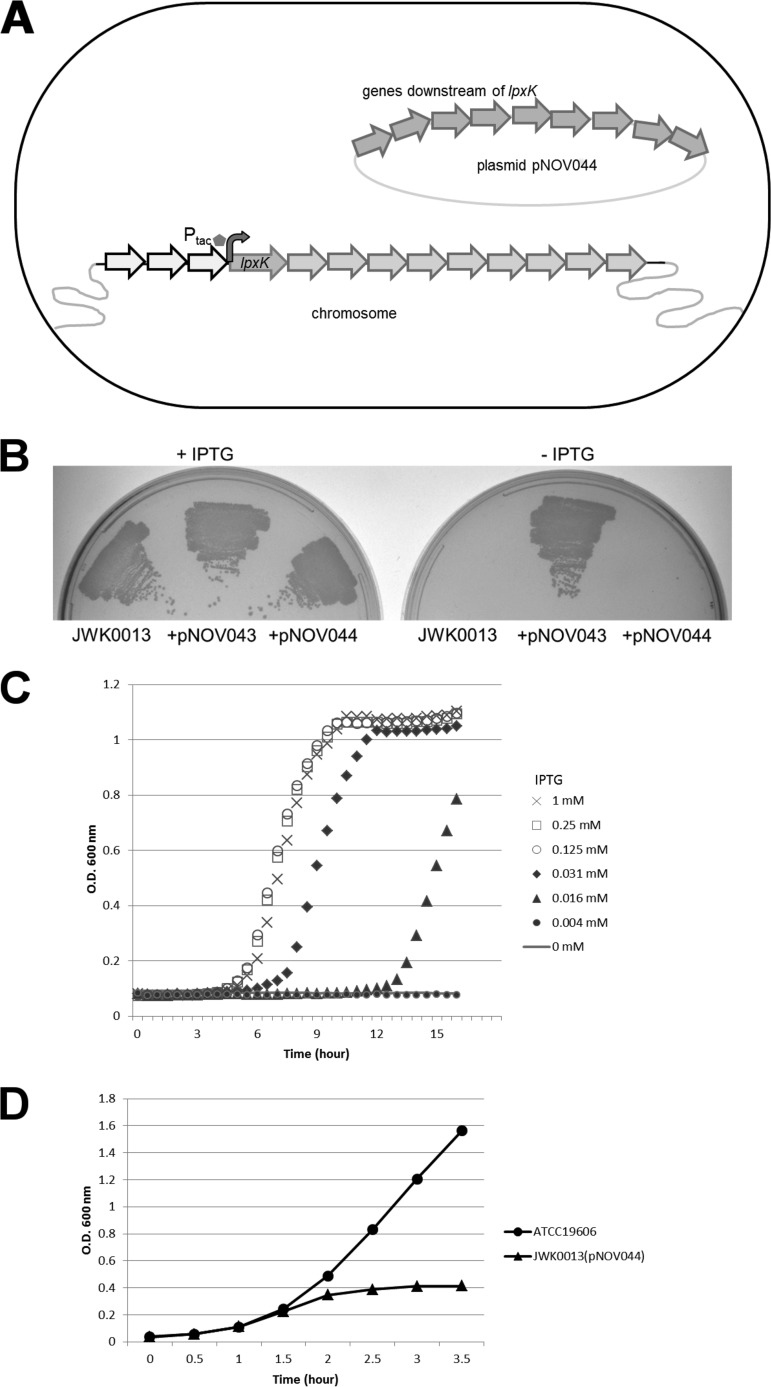
Schematic illustration of the *lpxK*-regulated strain JWK0013(pNOV044) and its dependence on LpxK expression for cell growth. (A) A P_tac_ promoter (inducible by IPTG) was inserted upstream of *lpxK* on the chromosome to create JWK0013. Plasmid pNOV044 contained the native *lpxK* promoter driving the cotranscribed genes downstream of *lpxK* to ensure the expression of these downstream genes in the absence of IPTG. (B) Growth of JWK0013 and JWK0013(pNOV044) is IPTG dependent, but growth of JWK0013(pNOV043) is not. (C) Growth curves of JWK0013(pNOV044) with various IPTG concentrations. (D) Representative growth curves of ATCC 19606 and JWK0013(pNOV044) for sample collection for microscopy in this study.

*A. baumannii* ATCC 19606 is capable of growth under standard laboratory conditions in the absence of LOS, and the LpxA, LpxC, and (possibly) LpxD enzymes that catalyze early steps of lipid A biosynthesis were shown to be dispensable for growth (11, 28). We recently demonstrated that LpxH, which catalyzes a step in lipid A biosynthesis downstream of these enzymes, is required for growth of *A. baumannii* ATCC 19606 under standard laboratory conditions. The loss of LpxH function led to toxic accumulation of detergent-like pathway intermediates (27). LpxK, an integral membrane kinase responsible for the phosphorylation of DSMP, has recently been proposed as an attractive target in the lipid A pathway, based on modeling predictions suggesting that it may catalyze the rate-limiting step in lipid A biosynthesis in *E. coli* (wherein LPS is essential [12]). To determine whether LpxK is also essential for growth in *A. baumannii* ATCC 19606, we attempted to delete *lpxK* on the genome. Attempts to delete *lpxK* via homologous recombination were unsuccessful, suggesting that LpxK was essential for growth in *A. baumannii* ATCC 19606. To confirm this, we first constructed an IPTG-inducible strain wherein the P_tac_ promoter and the *lacI* gene were inserted directly into the chromosome upstream of *lpxK* (strain JWK0013). However, in *A. baumannii* ATCC 19606, *lpxK* is the fourth gene within a predicted 13-gene operon containing multiple essential genes downstream of *lpxK* (e.g., encoding DNA polymerase III); therefore, any growth defect occurring in the absence of induction would not be attributable specifically to loss of LpxK. To deconvolute the determinants of such defects, we constructed 2 plasmids, pNOV043 and pNOV044; plasmid pNOV043 harbors a native promoter driving *lpxK* expression and includes all of the cotranscribed genes downstream of *lpxK* (and would therefore fully complement downregulation of the transcript in the absence of IPTG), and pNOV044 is identical to pNOV043 except that it lacks *lpxK* (and therefore harbors only the relevant genes located downstream of *lpxK*). Strain JWK0013(pNOV044) (depicted in Fig. 1A) thereby constitutes a specifically *lpxK*-regulated expression strain. When cultured on medium with or without IPTG, JWK0013 (no plasmid) and JWK0013(pNOV044) required IPTG for growth, whereas growth of JWK0013(pNOV043) was not IPTG dependent (Fig. 1B). This finding strongly supports the hypothesis that *lpxK* is essential for the growth of *A. baumannii* ATCC 19606. We confirmed this result in broth culture by demonstrating that growth of JWK0013(pNOV044) is dependent on IPTG in a dose-dependent manner (Fig. 1C).

### LpxK depletion caused morphological changes to cells.

**FIG 2:**
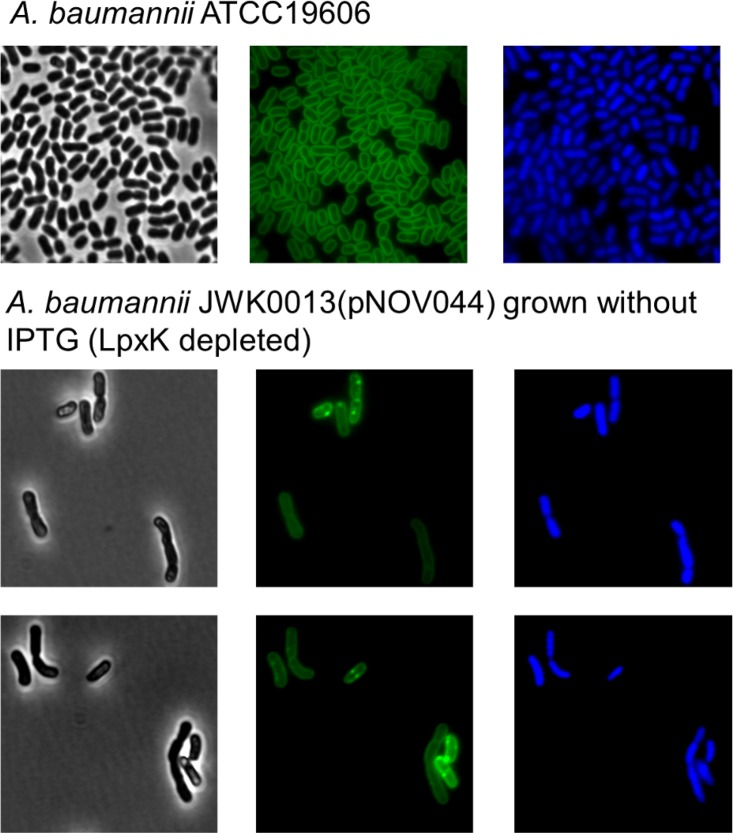
Fluorescence microscopy of *A. baumannii* cells depleted for LpxK. *A. baumannii* ATCC 19606 and JWK0013(pNOV044) were grown overnight with IPTG and then subcultured at an appropriate dilution into media without IPTG. Cells were collected after 3 h of growth (Fig. 1D) and fixed in 2% glutaraldehyde for microscopy. Green, membrane stained by FM1-43fx; blue, DNA stained by DAPI. All figures are to the same scale.

**FIG 3:**
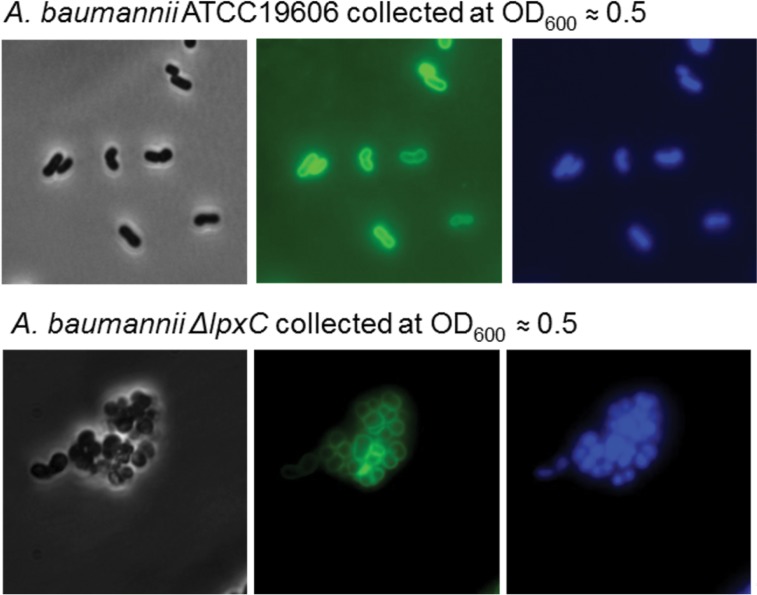
Fluorescence microscopic observation of *A. baumannii* Δ*lpxC* cells. *A. baumannii* ATCC 19606 and *A. baumannii* Δ*lpxC* grown to an OD_600_ of approximately 0.5 were fixed in 2% glutaraldehyde for microscopy. Green, membrane stained by FM1-43fx; blue, DNA stained by DAPI. The figures are all to the same scale.

**FIG 4:**
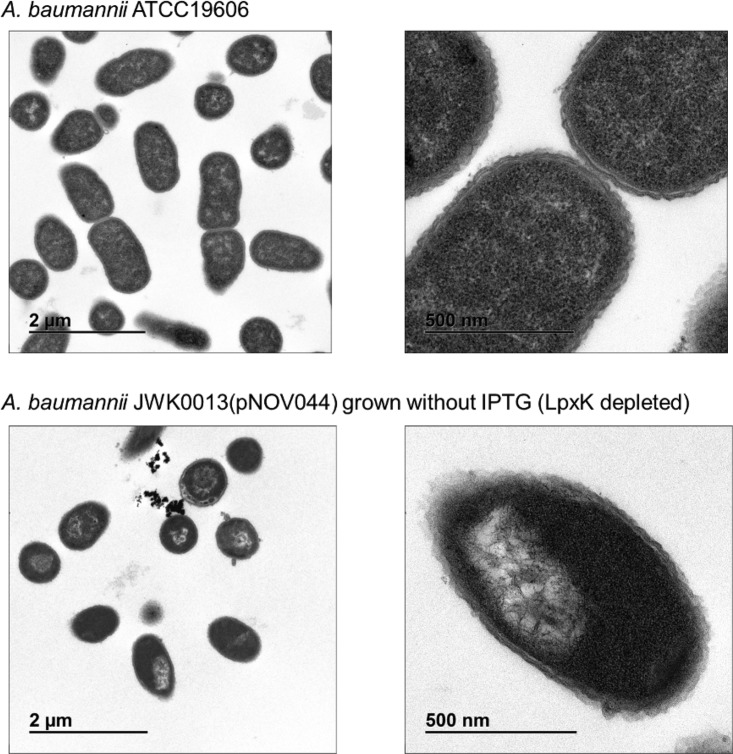
Transmission electron microscopy of *A. baumannii* cells depleted for LpxK. *A. baumannii* ATCC 19606 and JWK0013(pNOV044) were collected as described above for fluorescence microscopy (Fig. 2) and fixed with Tousimis fixative followed by observation using transmission electron microscopy.

To examine the effect of LpxK depletion on *A. baumannii* ATCC 19606 cellular morphology, we identified an appropriate inoculum of JWK0013(pNOV044) (1:8) to subculture into noninducing (−IPTG) medium so that progressive depletion of LpxK would reduce growth over time in liquid culture. We chose conditions (inocula) in which culture growth leveled off at an optical density at 600 nm (OD_600_) of approximately 0.4 to ensure that growth cessation could be clearly observed while providing an adequate quantity of cells for fluorescence and transmission electron microscopy (3 h time point of the growth curve in Fig. 1D). We also collected samples from the parental strain and from an *A. baumannii* Δ*lpxC* strain for comparison. Depletion of LpxK resulted in abnormal cell morphology, with elongated and bent cells compared to the parent strain observed by the use of fluorescence microscopy (Fig. 2). This differed from the phenotype of the Δ*lpxC* mutant, where cells were enlarged and rounded and clumped together (Fig. 3). Electron microscopy also revealed an aberrant inner membrane, blebbing of cell envelope, and increased vesicle formation in cells depleted for LpxK (Fig. 4). These apparent envelope defects are consistent with the notion of detergent-like lipid A pathway intermediates potentially accumulating in the inner membrane and causing its deformation by inducing curvature.

### Depletion of LpxK causes accumulation of LOS pathway intermediates in *A. baumannii* ATCC 19606.

**FIG 5:**
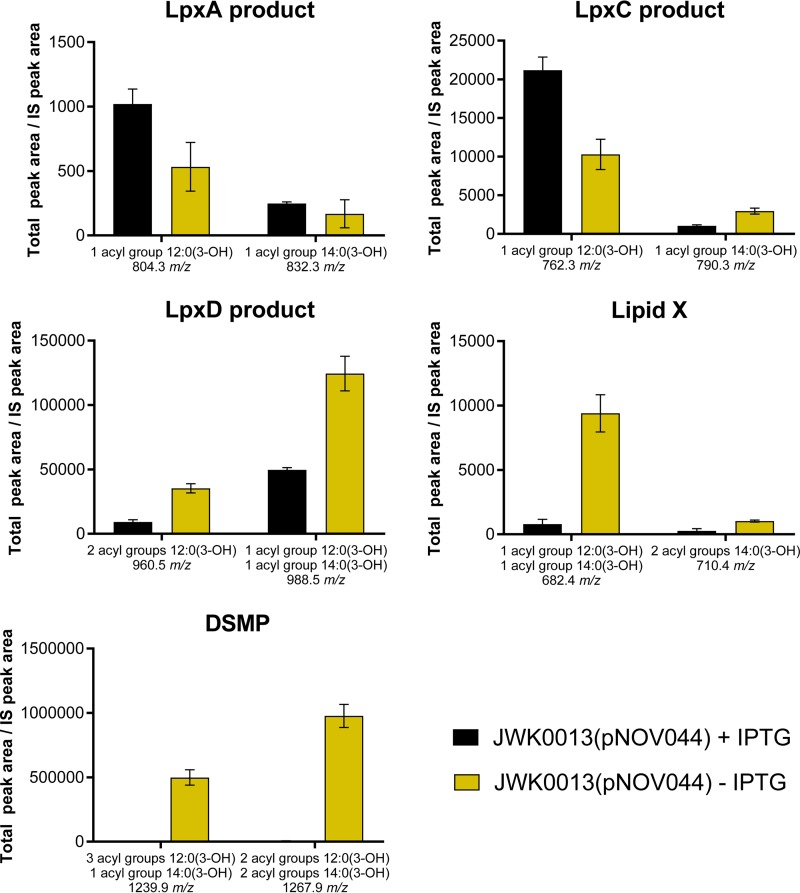
LCMS–multiple-reaction monitoring (LCMS-MRM) of lipid A pathway metabolites in JWK0013(pNOV044). The LCMS-MRM quantification of lipid A precursors from UDP-3-*O*-[(*R*)-3-OH-C_12/14_]-GlcNAc (LpxA product) through DSMP is shown for JWK0013(pNOV044) under inducing conditions (+IPTG) or noninducing conditions (−IPTG). Data are representative of results from three independent experiments performed in triplicate. The bars show mean values and standard deviations (SD). Data shown were normalized to an internal standard (IS) as previously described (27).

Depletion of LpxK affected bacterial growth and caused morphological changes to cells, possibly reflecting toxic accumulation of lipid A biosynthetic pathway intermediates. To test this hypothesis, we used LCMS to directly compare lipid A pathway intermediate levels for strain JWK0013(pNOV044) cultured with or without induction of LpxK expression by IPTG. For the noninduced sample, we subcultured JWK0013(pNOV044) (1:20) into noninducing (−IPTG) medium so that progressive depletion of LpxK would reduce growth over time in liquid culture while providing an adequate amount of cells for quantification of lipid A pathway intermediates (OD_600_, ~0.5). We observed significant accumulations of lipid A pathway intermediates, including DSMP and lipid X, in *A. baumannii* depleted for LpxK (Fig. 5). These findings supported our hypothesis that toxic accumulations may contribute to growth inhibition upon loss of LpxK function. Intriguingly, we also observed a small but reproducible decrease in the levels of LpxA and LpxC products (Fig. 5), despite the fact that these enzymes occur upstream of the block at LpxK. This suggested the possibility that *A. baumannii* ATCC 19606 may be able to decrease substrate flux into the lipid A biosynthetic pathway upon sensing certain toxic accumulations, but confirmation of this would require further exploration.

### Inhibition of LpxC rescues growth of cells depleted for LpxK and alleviates toxic intermediate accumulation.

**FIG 6:**
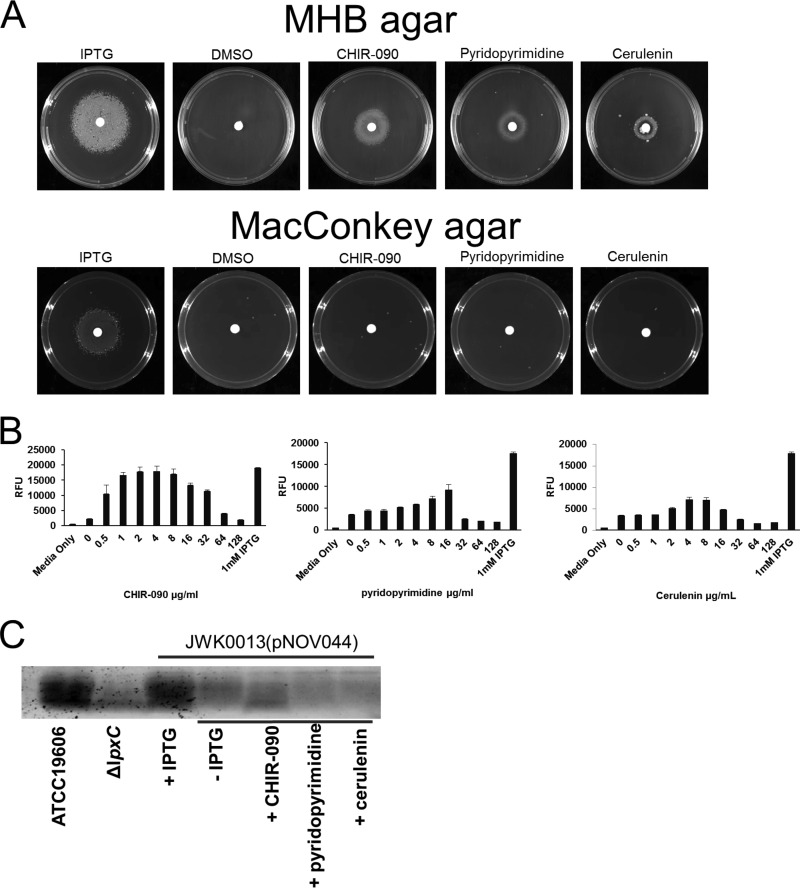
CHIR-090, cerulenin, and pyridopyrimidine can rescue the growth of LpxK-depleted cells. (A) JWK0013(pNOV044) was streaked on MHB agar supplemented with 1 mM IPTG and grown overnight at 37°C to induce LpxK expression. The following day, cells were washed repeatedly and resuspended to an OD_600_ of 0.01, and a 100-µl volume was plated on MHB agar or MacConkey agar plates without IPTG. Sterile filter discs spotted with IPTG, DMSO, CHIR-090, pyridopyrimidine, or cerulenin were placed on the plates, which were then incubated at 37°C for 24 h (cerulenin was incubated for 72 h). Growth of JWK0013(pNOV044) was restored in the presence of IPTG on both media. Growth of JWK0013(pNOV044) was not observed under noninducing conditions (minus IPTG and DMSO). JWK0013(pNOV044) grew under noninducing conditions in the presence of CHIR-090 (LpxC inhibitor), pyridopyrimidine (acetyl-CoA-carboxylase inhibitor), or cerulenin (β-ketoacyl-acyl carrier protein synthase inhibitor) on MHA but not MacConkey agar. (B) An overnight culture of JWK0013(pNOV044) grown under inducing conditions (+IPTG) was diluted to an OD_600_ of 0.1 and then was diluted 100-fold into MHB containing 10% alamarBlue. Next, 100 µl of the inoculum was added to the wells of a 96-well plate containing CHIR-090, pyridopyrimidine, or cerulenin to final assay concentrations ranging from 0 to 128 µg/ml. The plate was incubated for 6 h at 37°C before fluorescence (excitation, 545 nm; emission, 590 nm) was read on a SpectraMax microplate reader, and data were processed with Softmax Pro software v 5.4.1. (C) Cell-associated LOS levels during chemical growth rescue under conditions of LpxK depletion. Lane 1, *A. baumannii* ATCC 19606 (parent); lane 2, *A. baumannii lpxC*::*Km*^r^ (LOS-deficient cells); lane 3, *A. baumannii* JWK0013(pNOV044) grown with 1 mM IPTG; lane 4, *A. baumannii* JWK0013(pNOV044) harvested after grown in the absence of IPTG (LpxK-depleted cells); lane 5, *A. baumannii* JWK0013(pNOV044) grown in the absence of IPTG with 8 μg/ml CHIR-090; lane 6, *A. baumannii* JWK0013(pNOV044) grown in the absence of IPTG with 16 μg/ml pyridopyrimidine; lane 7, *A. baumannii* JWK0013(pNOV044) grown in the absence of IPTG with 8 μg/ml cerulenin. The LOS gel data are representative of results from three independent experiments.

**FIG 7:**
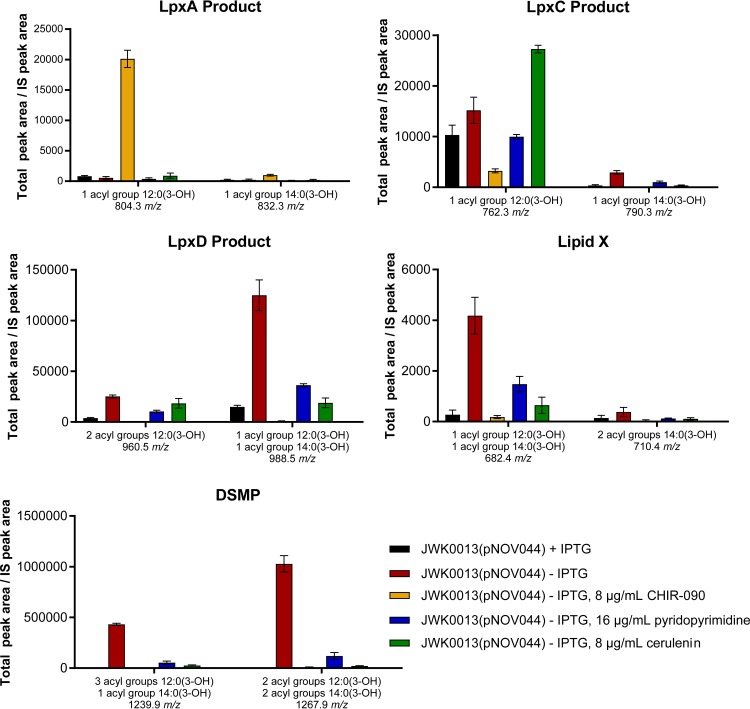
Effect of CHIR-090, cerulenin, or pyridopyrimidine on accumulation of lipid A pathway metabolites in JWK0013(pNOV044) determined by LCMS-MRM analysis. The LCMS-MRM quantification of lipid A precursors from UDP-3-*O*-[(*R*)-3-OH-C_12/14_]-GlcNAc (LpxA product) through DSMP is shown for JWK0013(pNOV044) under inducing conditions (+IPTG) and noninducing conditions (−IPTG) and/or in the presence of CHIR-090 at 8 µg/ml, pyridopyrimidine at 16 µg/ml, or cerulenin at 8 µg/ml. Experiments were performed in triplicate, and bars show means and SD. Data shown were normalized to an internal standard (IS) as previously described (27).

If the failure of JWK0013(pNOV044) to grow upon depletion of LpxK was indeed caused by the toxic accumulation of lipid A pathway intermediates, we would expect the growth of the strain to be restored by inhibiting LpxC. Supporting this idea, growth of JWK0013(pNOV044) was no longer IPTG dependent when the LpxC inhibitor CHIR-090 (29–32) was included in either solid growth medium (Fig. 6A, left side of the panel) or liquid cultures (Fig. 6B). Growth rescue was dose dependent, and it occurred at CHIR-090 concentrations shown previously (27) to dramatically reduce LOS production in *A. baumannii* ATCC 19606 (Fig. 6B and C). Similar growth rescue by CHIR-090 was seen when tryptic soy agar plates were used (data not shown). Furthermore, JWK0013(pNOV044) subcultured in the absence of IPTG under conditions (1:50 dilution) that required CHIR-090 (8 µg/ml) to grow and reach a culture OD_600_ of 0.5 accumulated much less DSMP and lipid X than the same strain grown under noninducing conditions using a 1:20 subculture dilution in the absence of CHIR-090, which reached an OD_600_ of 0.5 (Fig. 7). Therefore, the decrease in accumulation of detergent-like pathway intermediates occurred concomitantly with *A. baumannii* growth restoration mediated by inhibition of LpxC.

### Inhibitors of fatty acid biosynthesis rescue growth of cells depleted for LpxK and reduce accumulation of DSMP and lipid X.

A substantial body of work has demonstrated that there are important relationships between the fatty acid and lipid A biosynthetic pathways in Gram-negative bacteria. For example, in *E. coli*, *fabZ* mutations decrease susceptibility to LpxC inhibitors, and *accD* and *fabH* mutations can rescue the lethality of defects in the LPS transporter proteins LptF and LptG. We therefore tested whether the fatty acid biosynthesis (β-ketoacyl-acyl carrier protein synthase) inhibitor cerulenin (33) and the recently reported acetyl-CoA synthesis (biotin carboxylase, acetyl-CoA carboxylase [Acc]) inhibitor pyridopyrimidine (34) could rescue the growth of JWK0013(pNOV044) under noninducing conditions. Interestingly, growth of LpxK-depleted cells (−IPTG) was rescued by exposure to either compound, and the rescue effects were dose dependent, albeit they were not as robust as those observed for LpxC inhibition by CHIR-090 (Fig. 6A, right side of the panel). Similar growth rescue by these compounds was seen when tryptic soy agar plates were used (data not shown). Furthermore, JWK0013(pNOV044) subcultured at a dilution (1:50) that required cerulenin (8 µg/ml) or pyridopyrimidine (16 µg/ml) treatment to reach an OD_600_ of 0.5 had a significantly lower accumulation of DSMP and lipid X than the same strain under noninducing conditions using a 1:20 subculture dilution in the absence of either compound, which reached an OD_600_ of 0.5 (Fig. 7). The relative decrease in the levels of these metabolites was larger in CHIR-090-treated cells and smaller in cells treated with cerulenin or pyridopyrimidine. This suggests that inhibition of fatty acid biosynthesis by either of these compounds might also serve to reduce the toxic accumulation of pathway intermediates caused by loss of LpxK, restoring growth. It is of interest that when growth rescue is accomplished by blocking the early stages of lipid A biosynthesis with LpxC inhibitor CHIR-090, as described above, the growth-rescued cells would be expected to lack LOS and to not have an intact OM. Supporting this expectation, CHIR-090 could rescue cells on Mueller-Hinton broth (MHB) agar but not on MacConkey agar, which contains toxic bile salts, presumably reflecting a defect (lack) of an OM permeability barrier (Fig. 6A, bottom panel). Interestingly, growth rescue by cerulenin or pyridopyrimidine also occurred on MHB agar but not on MacConkey agar (Fig. 6A), indicating that although growth *per se* could be restored, the cells apparently still exhibited a permeability defect. Possibly explaining this, cell-associated LOS in the chemically rescued cells was not restored to levels comparable to those seen with wild-type ATCC 19606 or the LpxK-regulated strain [JWK0013(pNOV044)] grown in the presence of IPTG, further suggesting that the OM was still defective in the chemically rescued cells (Fig. 6C).

## DISCUSSION

Identification of bacterial proteins whose functional inhibition can cause toxic accumulation of pathway intermediates could inform selection of antibacterial targets. For example, although lipid A biosynthesis itself is essential *per se* in organisms such as *E. coli*, an enzyme such as LpxD, within the lipid A biosynthetic pathway, has been implicated as a particularly good candidate antibacterial target since its substrate is detergent-like and therefore cells may be particularly sensitive to inhibition of LpxD (35). However, whether toxic accumulations are playing a role in growth inhibition is difficult to fully explore when the product of the pathway itself (e.g., lipid A) is essential for growth. The ability of *A. baumannii* ATCC 19606 to grow without lipid A biosynthesis, which can be caused by inactivation of genes encoding enzymes catalyzing early steps of the pathway (e.g., *lpxC*), provides an elegant platform with which to explore the potential role of toxic intermediate accumulation in mediating the essentiality of certain catalytic steps that occur later in the lipid A biosynthetic pathway. We previously demonstrated, using a strain with controlled expression of *lpxH*, that LpxH was essential for growth in *A. baumannii* ATCC 19606 due to this phenomenon. Here we extend this finding to include LpxK, which acts later in the pathway than LpxH (see Fig. S4 in the supplemental material). LpxK has also drawn recent interest as a potential antibacterial target since it may be represent a rate-limiting step in lipid A biosynthesis (12, 13). Here we show that *A. baumannii* ATCC 19606 cells depleted for LpxK fail to grow, suggesting that inhibitors of LpxK may be antibacterial for strains where LOS is not essential, such as *A. baumannii* ATCC 19606. Therefore, the antibacterial potency of LpxK inhibitors should be measurable using routine antimicrobial susceptibility testing protocols, such as those outlined by the Clinical and Laboratory Standards Institute (CLSI; M100, 27th edition).

The accumulation of DSMP and lipid X observed upon depletion of LpxK strongly indicates that toxic accumulation(s) plays a role in the growth phenotypes of depleted strains. The ability of the LpxC inhibitor CHIR-090 to rescue growth of cells downregulated for LpxK strongly supports this hypothesis. In the case of *A. baumannii* ATCC 19606, our observations explain why LpxK (and, previously, LpxH) is essential for growth. In Gram-negative pathogens where LPS itself is essential (e.g., *E. coli* or *P. aeruginosa*), such toxic accumulations could mean that bacteria could be particularly sensitive to inhibition of targets such as LpxK due to the contribution of both inhibition of lipid A synthesis and toxic precursor accumulation.

Intriguingly, we also observed that exposure of LpxK-depleted *A. baumanni* ATCC 19606 to inhibitors of fatty acid biosynthesis (cerulenin and pyridopyrimidine) rescued the growth defect caused by loss of LpxK and reduced the accumulation of DSMP and lipid X. The fatty acid biosynthetic pathway is necessary for biosynthesis of both the inner membrane (phospholipid bilayer) and the OM (phospholipid inner leaflet and LPS outer leaflet) and in organisms such as *E. coli*, where the balance of the levels of biogenesis of these membranes is crucial for bacterial growth. This is further reflected in the observation that mutations in fatty acid genes (*fabZ* in *E. coli* [36] and *fabG* in *P. aeruginosa* [37]) decreased susceptibility to LpxC inhibitors, which was proposed to result from increased substrate flux into the lipid A pathway (38). A reduction in LpxC levels was observed in *E. coli fabZ* mutants, suggesting a regulatory relationship between the pathways (36). Presumably, strains like *A. baumannii* ATCC 19606 would also need to balance phospholipid (PL) and LOS levels under normal conditions when LOS is being made, so as to generate a proper cell envelope and to maintain the asymmetric permeability barrier of the OM. The absence of PBP1A and the corresponding upregulation of several surface-associated lipoproteins were shown to underlie the ability of strains like *A. baumannii* ATCC 19606 to tolerate complete loss of lipid A (25), but how the cells respond to lipid A loss in terms of fatty acid biosynthesis remains to be elucidated (11, 39, 40). The impact of inhibiting fatty acid synthesis in reversing the LpxK dependency of *A. baumannii* ATCC 19606, demonstrated here, may mainly reflect relief of toxic lipid A precursor accumulation, since rescued cells show a significant reduction of DSMP and lipid X accumulation concomitant with growth restoration, but this remains to be conclusively shown. However, growth rescue of LpxK-depleted cells via inhibition of either fatty acid biosynthesis or LpxC was successful only when cells were grown on typical laboratory media such as Mueller-Hinton agar (MHA) but not MacConkey agar, suggesting that the rescued cells lack a functional OM permeability barrier. Supporting this, cell-associated LOS levels were not restored to normal during chemical rescue. This is expected in the case of LpxC inhibition, which blocks lipid A synthesis at an early stage, making it highly unlikely that a normal OM could be reestablished in the growth-rescued cells. The inability to grow on MacConkey agar and reduced cell-associated LOS levels seen in cells rescued by inhibition of fatty acid biosynthesis suggest that growth rescue in that case may reflect a reduction of substrate flow into lipid A biosynthesis. Intriguingly, *accD* and *fabH* mutations were able to suppress the lethality of an *E. coli* strain with mutations affecting LptF and LptG (41), which mediate transport of LPS to the OM. The suppression of lethality of the *E. coli lptFG* mutation by *accD* and *fabH* mutations might also involve a reduction of toxic intermediates. Since *E. coli* requires LPS for growth, these mutations may reduce substrate flow into the lipid A pathway, first relieving (preventing) the toxic accumulation and ultimately reestablishing the balance between PL and LPS levels according to the residual rate of LPS transport mediated by the partially defective LptFG transport proteins. In contrast to what we have observed here, those suppressor mutants can have an intact OM permeability barrier, suggesting proper restoration of an LPS-containing OM (41). The exact mechanism by which inhibition of fatty acid biosynthesis mediates growth rescue in *A. baumannii* remains to be fully elucidated, but our data suggest that reduction of the accumulation of toxic intermediates is at least partly responsible.

Finally, it may be useful to identify nodes of antibacterial synergy within essential biosynthetic pathways, as exemplified by the synergy seen for trimethoprim-sulfamethoxazole treatments, which inhibit the dihydrofolate reductase and dihydropteroate synthetase enzymes in the folate biosynthesis pathway (42). Here we have explored the potential of toxic intermediate accumulation in the context of inhibiting individual targets within the lipid A biosynthetic pathway. However, pathways having potentially toxic intermediates may also be intrinsically subject to growth rescue by relief of this accumulation, as shown here and elsewhere, which could be observed as antagonism between inhibitors of certain individual targets. In the case of *A. baumannii* ATCC 19606, this may be less of an issue for the lipid A pathway, since growth-rescued cells appear to have highly defective envelopes, but this revives the notion that the characterization of potential synergies in biosynthetic pathways must be carefully and systematically undertaken.

## MATERIALS AND METHODS

### Bacterial strains, plasmids, and growth conditions.

The bacterial strains and plasmids used in this study are listed in Table 1. *A. baumannii* ATCC 19606 was purchased from the American Type Culture Collection (ATCC), and JWK0013(pNOV044), an IPTG-regulated *lpxK* strain, was constructed as described below. The oligonucleotides used in this study are listed in Table S1. Cells were routinely grown in cation-adjusted Mueller-Hinton broth (MHB) (3.0 g/liter beef extract, 17.5 g/liter acid hydrolysate of casein, 1.5 g/liter starch, 20 to 25 mg/liter calcium, 10 to 12.5 mg/liter magnesium) or tryptic soy broth (TSB) (17.0 g/liter pancreatic digest of casein, 3.0 g/liter peptic digest of soy bean meal, 2.5 g/liter dextrose, 5.0 g/liter sodium chloride, 2.5 g/liter dipotassium hydrogen-phosphate). Lysogeny broth (LB) was used for routine growth of *E. coli* (10 g/liter tryptone, 5 g/liter yeast extract, 10 g/liter NaCl). Gentamicin (10 µg/ml for *E. coli* or 100 µg/ml for *A. baumannii*), kanamycin (50 µg/ml), and IPTG (up to 1 mM) were added when necessary. The LpxC inhibitor CHIR-090 was described previously (37). The β-ketoacyl-acyl carrier protein synthase inhibitor cerulenin (33) was purchased from Sigma, and pyridopyrimidine, an acetyl-CoA-carboxylase inhibitor (34), was synthesized at Novartis.

10.1128/mSphere.00199-17.2 TABLE S1  Sequences of oligonucleotide primers used in this study. Download TABLE S1, DOCX file, 0.02 MB. Copyright © 2017 Wei et al. 2017 Wei et al. This content is distributed under the terms of the Creative Commons Attribution 4.0 International license.

**TABLE 1:** Bacterial strains and plasmids used in this study

Straink or plasmid	Relevant characteristic(s)	Reference or source
Strains		
*A. baumannii* ATCC 19606	Wild-type strain	American Type Culture Collection (ATCC)
*A. baumannii* JWK0013	*A. baumannii* ATCC 19606 derivative, Ptac::*lpxK* (Km^r^), *lacI*-Ptac inserted in front of *lpxK*	This study
*A. baumannii* Δ*lpxC* mutant (NB48062-LMD0007)	*A. baumannii* ATCC 19606 derivative* lpxC* mutant, *lpxC*::*aph*(Km^r^)	45
Plasmids		
pC009	pUC19 vector harboring an *sspB*::*aph*(Km^r^) integration cassette	27
pNOV002	pBR322 ori, pWH1266, Ptac::*gfp*, *bla*	27
pNOV018	pBR322 ori, pWH1266, PKm::*lacI*, *aacC1*(Gm^r^)^a^	27
pNOV043	pBR322 ori, pWH1266, native promoter driving *lpxK* and genes downstream of *lpxK*	This study
pNOV044	pBR322 ori, pWH1266, native promoter driving genes downstream of *lpxK*	This study

a Gm^r^, gentamicin resistance.

### Construction of IPTG-inducible LpxK strain JWK0013(pNOV044) in *A. baumannii* ATCC 19606.

To make the *lpxK* gene on the chromosome of *A. baumannii* ATCC 19606 regulated by IPTG, the P_tac_ promoter and *lacI* were inserted in front of the *lpxK* gene on the chromosome to generate strain JWK0013. The upstream region of *lpxK* was amplified from strain *A. baumannii* ATCC 19606 genomic DNA using primers KTT433 and KTT434; KTT434 attaches a linker to the *aph*(Km^r^) (*aph* kanamycin resistance) cassette on the 3′ end of the fragment. The *aph*(Km^r^) cassette was amplified using primers KTT85 and KTT86 from plasmid pC009. The *lacI* gene and P_tac_ promoter were amplified from pNOV002 using primers KTT238 and KTT239; KTT239 attaches a linker to the *aph*(Km^r^) cassette on the 5′ end of the fragment. The *lpxK* gene was amplified from *A. baumannii* ATCC 19606 genomic DNA using primers KTT435, which incorporates a linker to the P_tac_ promoter on the 5′ end of the fragment, and KTT436. The final construct was generated by overlap extension PCR (43) to ligate the upstream fragment, the *aph*(Km^r^) cassette, the *lacI* gene and P_tac_ fragment, and the downstream fragment. The resultant DNA constructs were then transformed into *A. baumannii* ATCC 19606 via electroporation. One of the resulting transformants, JWK0013, was confirmed for correct integration of the constructs by PCR and sequencing using primers cPCR lpxK F and cPCR lpxK R.

Plasmid pNOV043 was generated by cloning the promoter of the putative operon containing the *lpxK* gene (*lpxK* is the fourth gene of the operon) and the genes downstream of *lpxK* into the backbone generated from pNOV018, which contains the pBR322 ori, pWH1266, and P_Km_::*lacI* and the *aacC1* gentamicin resistance cassette. Plasmid pNOV044 is similar to pNOV043 except that it lacks the *lpxK* gene. Both plasmids are deposited in GenBank (see below). The primers used to construct pNOV043 and pNOV044 are listed in Table S1 in the supplemental material. The vectors were verified by sequencing and were electroporated into JWK0013 to generate JWK0013(pNOV043) and JWK0013(pNOV044) (Fig. 1B).

### Growth curve of JWK0013(pNOV044) with different concentrations of IPTG.

JWK0013(pNOV044) (−80°C) was streaked onto an LB plate with gentamicin and IPTG. The following day, a bacterial suspension was prepared using a BBL prompt inoculation system (Becton, Dickinson and Company, Franklin Lakes, NJ) and further diluted 1:1,000 in MHB. In a 96-well flat-bottom plate, IPTG was serially diluted in MHB and the diluted bacterial suspension was applied to the serially diluted IPTG solution. The plate was incubated in a Spectramax detection platform (Softmax Pro software v 5.4.1) at 37°C with shaking, and the contents were measured every minute. The results were then analyzed and plotted in Excel (Fig. 1C).

### Monitoring cellular morphology under conditions of LpxK depletion.

The *A. baumannii* ATCC 19606 parental strain, the *A. baumannii* Δ*lpxC* mutant, and JWK0013(pNOV044) were grown overnight in MHB. Strain JWK0013(pNOV044) was grown overnight in MHB with 50 µg/ml gentamicin and 1 mM IPTG for induction of LpxK expression. The cells were diluted 1:100 the next day in 50 ml MHB (*A. baumannii* ATCC 19606 or *A. baumannii ΔlpxC*) or 50 ml MHB–1 mM IPTG [JWK0013(pNOV044)] and grown at 37°C with shaking. When the culture reached an OD_600_ of ~0.5, cells were collected by centrifugation, washed twice with MHB, suspended in same volume of MHB, and then diluted to 1:8 in 300 ml MHB in a 1-liter flask. These cultures were grown at 37°C with shaking, and samples were collected every 30 min for OD_600_ measurement, CFU counting, and microscopy. Figure 1D shows the representative growth curve determined by OD_600_ measurement. For fluorescence microscopy, the cells were collected and 25% glutaraldehyde (Sigma G6257) was added to reach a final concentration of 2%. The cells were fixed on 1.2% agar on the slide, stained with either 100 µg/ml 4′,6-diamidino-2-phenylindole (DAPI) (catalog no. 62248; Thermo Scientific) or 10 µg/ml FM1-43fx (catalog no. F35355; Life Technologies, Inc.), and observed using a Nikon Eclipse Ti inverted microscope with a Nikon halogen illuminator (D-LH/LC), a Sola light engine (Lumencor, Beaverton, OR), and a Clara Interline charge-coupled-device (CCD) camera (Andor, South Windsor, CT). A Nikon CFI Plan Apo Lamda DM ×100 oil objective lens (1.45 numerical aperture [NA]) was used for phase-contrast and fluorescence imaging. For FM 1-43fx images, we used a fluorescein isothiocyanate (FITC)-5050ANTE-ZERO filter set (Semrock, Rochester, NY). The DAPI images were taken by using a BFP-A-Basic-NTE filter set (Semrock). The exposure times for DAPI images and green fluorescent protein (GFP) images were 500 ms and 100 ms, respectively. Images were captured by using Nikon Elements software and exported for figure preparation in ImageJ (44). For thin-section transmission electron microscopy, cells were pelleted by centrifugation at 4,000 × *g* for 10 min at room temperature and then resuspended and fixed with Tousimis fixative (1.5% glutaraldehyde–1% formic acid–0.12 M Sorensen’s buffer) from Tousimis Research Corporation. The samples were submitted for embedding and thin-section preparation to the Center for Biophotonics Science & Technology of the University of California, Davis (Sacramento, CA, USA).

### Liquid chromatography-mass spectrometry (LCMS) detection of lipid A precursors.

*A. baumannii* IPTG-regulated *lpxK* strain JWK0013(pNOV044) was grown overnight in MHB supplemented with 1 mM IPTG to induce LpxK expression. The following day, the cells were diluted to an OD_600_ of 0.05 in 50 ml of MHB–1 mM IPTG and grown at 37°C with shaking until an OD_600_ of 0.5 was reached. Cells were then collected by centrifugation, washed twice with MHB, and suspended in MHB at the original volume of 50 ml. The cell suspension was then diluted 1:20 in 50 ml of fresh MHB with or without IPTG in 250-ml flasks. The cultures were grown at 37°C with shaking, and samples were collected every hour for OD_600_ measurement. When the cultures reached an OD_600_ of 0.5, a 5-ml volume was removed and frozen at −80°C. For the rescue experiments, *A. baumannii* JWK0013(pNOV044) was again grown overnight in MHB supplemented with 1 mM IPTG for induction of LpxK expression. The cells were diluted in 50 ml MHB–1 mM IPTG to an OD_600_ of 0.05 the next day and grown at 37°C with shaking until the OD_600_ reached 0.5. When the culture reached an OD_600_ of ~0.5, cells were collected by centrifugation, washed twice with MHB, and suspended in same volume of MHB and then diluted to 1:50 in MHB, and 10 ml was added to a 50-ml conical tube with 1 mM IPTG, 8 µg/ml CHIR-090, 16 µg/ml pyridopyrimidine, or 8 µg/ml cerulenin. These cultures were grown at 37°C with shaking (220 rpm), and samples were collected for OD_600_ measurement. Once the cultures reached an OD_600_ of ~0.5 (after ~2 h 30 min for IPTG, ~3 h 15 min for CHIR-090, ~5 h for pyridopyrimidine, and ~7 h for cerulenin), the sample OD_600_ was adjusted to 0.5, a 5-ml volume was removed and placed in a 15-ml Falcon tube and centrifuged at 10,000 rpm, and the supernatant was removed and the pellet placed at −80°C until LCMS could be performed. Each experiment was performed in triplicate at least three times. LCMS analyses were done as described previously (27, 45), and data were analyzed in Skyline (46). The two-tailed Student’s *t* test was used for statistical analysis. The predicted lipid A biosynthetic pathway in *A. baumannii* ATCC 19606 with corresponding *m/z* values is shown in Fig. S1.

10.1128/mSphere.00199-17.1 FIG S1  Predicted lipid A biosynthetic pathway in *A. baumannii* ATCC 19606. Download FIG S1, TIF file, 1.2 MB. Copyright © 2017 Wei et al. 2017 Wei et al. This content is distributed under the terms of the Creative Commons Attribution 4.0 International license.

### Growth rescue of JWK0013(pNOV044) by the LpxC and fatty acid biosynthesis inhibitors under noninducing conditions.

To determine if inhibiting LpxC or fatty acid biosynthesis could rescue growth during LpxK depletion, JWK0013(pNOV044) was grown overnight at 37°C on MHA supplemented with 1 mM IPTG (Calbiochem). The following day, cells were suspended in 1 ml of MHB (1.5-ml microcentrifuge tube), collected by centrifugation, and suspended in fresh MHB for a total of 3 washes to remove trace amounts of IPTG. After the final wash, cells were suspended in 5 ml of MHB and the OD_600_ was adjusted to 0.01. Next, 100 µl of the cell suspension was spread on a fresh MHA plates and allowed to dry. Sterile paper disks (Remel; catalog no. R55054) were added to the center of the plates and inoculated with 10 μl of dimethyl sulfoxide (DMSO) (Sigma), CHIR-090 (LpxC inhibitor [29]; 12.8 mg/ml), cerulenin (β-ketoacyl-acyl carrier protein synthase inhibitor [33]; 12.8 mg/ml), or pyridopyrimidine (acetyl-CoA-carboxylase inhibitor [34]; 12.8 mg/ml). The plates were incubated at 37°C for 24 to 72 h before images were taken. Images were taken by the use of a Bio-Rad Universal Hood III system with Image Lab v 5.1 software. This procedure was repeated on MacConkey agar.

To establish the absolute concentrations of CHIR-090, pyridopyrimidine, and cerulenin that rescue growth of JWK0013(pNOV044), a broth-based assay was used. Compounds were dissolved in DMSO at 12.8 mg/ml (a concentration 100-fold higher than the final assay top concentration of 128 µg/ml). In a 96-well plate, sequential 2-fold serial dilutions of the content of wells 3 to 11 were made in DMSO (corresponding final assay concentrations of 0.5 to 128 µg/ml), leaving well 1 as the medium-only control, well 2 as the DMSO vehicle control, and well 12 for the IPTG (1 mM final concentration) growth control. Using a 12-channel electronic pipette, 1 µl of each 100× drug concentration, including the DMSO-only control, was transferred into a new 96-well U-bottom plate (Greiner Bio-One; catalog no. 650162). Next, using an 8-channel electronic pipette, the drug dilutions were then diluted 100-fold with the overnight inoculum for 6 to 8 h at 37°C before fluorescence reading was performed (excitation, 545 nm; emission, 590 nm) on a SpectraMax detection platform with Softmax Pro software v 5.4.1. To generate the overnight culture, JWK0013(pNOV044) was grown overnight in MHB with 1 mM IPTG for induction of LpxK expression. The following day, the cells were diluted to an OD_600_ of 0.1 and a further 100-fold in fresh MHB media with 10% alamarBlue (Bio-Rad). The experiment was done in triplicate.

### LOS quantity determined by gel electrophoresis.

For sample collection, *A. baumannii* JWK0013(pNOV044) was grown overnight in MHB–1 mM IPTG for induction of LpxK expression. The cells were diluted the next day to an OD_600_ of 0.05 in 50 ml MHB–1 mM IPTG and grown at 37°C with shaking for 2.5 h in a 250-ml flask until the OD_600_ reached 0.5. When the culture reached an OD_600_ of ~0.5, cells were collected by centrifugation, washed twice with MHB, and suspended in same volume of MHB and then diluted to 1:50 in 5 ml MHB in a 50-ml conical tube with or without IPTG and 8 µg/ml CHIR-090, 16 µg/ml pyridopyrimidine, or 8 µg/ml cerulenin. To obtain the same cell mass, the culture without IPTG alone (without rescuing chemicals) was diluted to 1:20. These cultures were grown at 37°C with shaking (220 rpm), and samples were collected for OD_600_ measurement. Once the cultures reached an OD_600_ of ~0.5, the samples were collected in a microcentrifuge tube. The tube was spun down at 10,000 rpm, the supernatant was removed, and the pellet was placed at −80°C until an LOS gel experiment could be performed as previously described (45).

### Accession number(s).

The sequences of the pNOV043 and pNOV044 plasmids were deposited in GenBank under accession numbers KY933087 (pNOV043) and KY933088 (pNOV044).

## References

[1] Antunes LC, Visca P, Towner KJ 2014 *Acinetobacter* *baumannii*: evolution of a global pathogen. Pathog Dis 71:292–301. doi:10.1111/2049-632X.12125. 24376225

[2] Gonzalez-Villoria AM, Valverde-Garduno V 2016 Antibiotic-resistant *Acinetobacter* *baumannii* increasing success remains a challenge as a nosocomial pathogen. J Pathog 2016:7318075. doi:10.1155/2016/7318075. PMC4757776 26966582

[3] Zavascki AP, Carvalhaes CG, Picão RC, Gales AC 2010 Multidrug-resistant *Pseudomonas aeruginosa* and *Acinetobacter* *baumannii*: resistance mechanisms and implications for therapy. Expert Rev Anti Infect Ther 8:71–93. doi:10.1586/eri.09.108. 20014903

[4] Gordon NC, Wareham DW 2010 Multidrug-resistant *Acinetobacter* *baumannii*: mechanisms of virulence and resistance. Int J Antimicrob Agents 35:219–226. doi:10.1016/j.ijantimicag.2009.10.024. 20047818

[5] Manchanda V, Sanchaita S, Singh N 2010 Multidrug resistant Acinetobacter. J Glob Infect Dis 2:291–304. doi:10.4103/0974-777X.68538. 20927292 PMC2946687

[6] Bos MP, Robert V, Tommassen J 2007 Biogenesis of the gram-negative bacterial outer membrane. Annu Rev Microbiol 61:191–214. doi:10.1146/annurev.micro.61.080706.093245. 17506684

[7] Weber BS, Harding CM, Feldman MF 2015 Pathogenic Acinetobacter: from the cell surface to infinity and beyond. J Bacteriol 198:880–887. doi:10.1128/JB.00906-15. 26712938 PMC4772598

[8] Brown DG 2016 Drug discovery strategies to outer membrane targets in Gram-negative pathogens. Bioorg Med Chem 24:6320–6331. doi:10.1016/j.bmc.2016.05.004. 27178386

[9] Piet JR, Zariri A, Fransen F, Schipper K, van der Ley P, van de Beek D, van der Ende A 2014 Meningitis caused by a lipopolysaccharide deficient *Neisseria meningitidis*. J Infect 69:352–357. doi:10.1016/j.jinf.2014.06.005. 24932738

[10] Lin L, Tan B, Pantapalangkoor P, Ho T, Baquir B, Tomaras A, Montgomery JI, Reilly U, Barbacci EG, Hujer K, Bonomo RA, Fernandez L, Hancock RE, Adams MD, French SW, Buslon VS, Spellberg B 2012 Inhibition of LpxC protects mice from resistant *Acinetobacter* *baumannii* by modulating inflammation and enhancing phagocytosis. mBio 3:e00312-12. doi:10.1128/mBio.00312-12. PMC3518917 23033474

[11] Moffatt JH, Harper M, Harrison P, Hale JD, Vinogradov E, Seemann T, Henry R, Crane B, St Michael F, Cox AD, Adler B, Nation RL, Li J, Boyce JD 2010 Colistin resistance in *Acinetobacter* *baumannii* is mediated by complete loss of lipopolysaccharide production. Antimicrob Agents Chemother 54:4971–4977. doi:10.1128/AAC.00834-10. 20855724 PMC2981238

[12] Emiola A, George J, Andrews SS 2014 A complete pathway model for lipid A biosynthesis in *Escherichia coli*. PLoS One 10:e0121216. doi:10.1371/journal.pone.0121216. 25919634 PMC4412817

[13] Emiola A, Andrews SS, Heller C, George J 2016 Crosstalk between the lipopolysaccharide and phospholipid pathways during outer membrane biogenesis in *Escherichia coli*. Proc Natl Acad Sci U S A 113:3108–3113. doi:10.1073/pnas.1521168113. 26929331 PMC4801286

[14] Babinski KJ, Kanjilal SJ, Raetz CR 2002 Accumulation of the lipid A precursor UDP-2,3-diacylglucosamine in an *Escherichia coli* mutant lacking the *lpxH* gene. J Biol Chem 277:25947–25956. doi:10.1074/jbc.M204068200. 12000771

[15] Babinski KJ, Ribeiro AA, Raetz CR 2002 The *Escherichia coli* gene encoding the UDP-2,3-diacylglucosamine pyrophosphatase of lipid A biosynthesis. J Biol Chem 277:25937–25946. doi:10.1074/jbc.M204067200. 12000770

[16] ‘Garrett TA, Kadrmas JL, Raetz CR 1997 Identification of the gene encoding the Escherichia coli lipid A 4’-kinase. Facile phosphorylation of endotoxin analogs with recombinant LpxK. J Biol Chem 272:21855–21864. doi:10.1074/jbc.272.35.21855. 9268317

[17] Doerrler WT, Gibbons HS, Raetz CR 2004 MsbA-dependent translocation of lipids across the inner membrane of *Escherichia coli*. J Biol Chem 279:45102–45109. doi:10.1074/jbc.M408106200. 15304478

[18] Doerrler WT, Raetz CR 2002 ATPase activity of the MsbA lipid flippase of *Escherichia coli*. J Biol Chem 277:36697–36705. doi:10.1074/jbc.M205857200. 12119303

[19] Whitfield C, Trent MS 2014 Biosynthesis and export of bacterial lipopolysaccharides. Annu Rev Biochem 83:99–128. doi:10.1146/annurev-biochem-060713-035600. 24580642

[20] Wang X, Quinn PJ 2010 Lipopolysaccharide: biosynthetic pathway and structure modification. Prog Lipid Res 49:97–107. doi:10.1016/j.plipres.2009.06.002. 19815028

[21] Klein G, Lindner B, Brabetz W, Brade H, Raina S 2009 *Escherichia coli* K-12 suppressor-free mutants lacking early glycosyltransferases and late acyltransferases: minimal lipopolysaccharide structure and induction of envelope stress response. J Biol Chem 284:15369–15389. doi:10.1074/jbc.M900490200. 19346244 PMC2708834

[22] Walsh AG, Matewish MJ, Burrows LL, Monteiro MA, Perry MB, Lam JS 2000 Lipopolysaccharide core phosphates are required for viability and intrinsic drug resistance in *Pseudomonas aeruginosa*. Mol Microbiol 35:718–727. doi:10.1046/j.1365-2958.2000.01741.x. 10692150

[23] Zhao X, Lam JS 2002 WaaP of *Pseudomonas aeruginosa* is a novel eukaryotic type protein-tyrosine kinase as well as a sugar kinase essential for the biosynthesis of core lipopolysaccharide. J Biol Chem 277:4722–4730. doi:10.1074/jbc.M107803200. 11741974

[24] Delucia AM, Six DA, Caughlan RE, Gee P, Hunt I, Lam JS, Dean CR 2011 Lipopolysaccharide (LPS) inner-core phosphates are required for complete LPS synthesis and transport to the outer membrane in *Pseudomonas aeruginosa* PAO1. mBio 2:e00142-11. doi:10.1128/mBio.00142-11. 21810964 PMC3147165

[25] Boll JM, Crofts AA, Peters K, Cattoir V, Vollmer W, Davies BW, Trent MS 2016 A penicillin-binding protein inhibits selection of colistin-resistant, lipooligosaccharide-deficient *Acinetobacter* *baumannii*. Proc Natl Acad Sci U S A 113:E6228–E6237. doi:10.1073/pnas.1611594113. 27681618 PMC5068286

[26] Radika K, Raetz CR 1988 Purification and properties of lipid A disaccharide synthase of *Escherichia coli*. J Biol Chem 263:14859–14867. 3049593

[27] Richie DL, Takeoka KT, Bojkovic J, Metzger LEt, Rath CM, Sawyer WS, Wei JR, Dean CR 2016 Toxic accumulation of LPS pathway intermediates underlies the requirement of LpxH for growth of *Acinetobacter* *baumannii* ATCC 19606. PLoS One 11:e0160918. doi:10.1371/journal.pone.0160918. 27526195 PMC4985137

[28] Moffatt JH, Harper M, Adler B, Nation RL, Li J, Boyce JD 2011 Insertion sequence ISAba11 is involved in colistin resistance and loss of lipopolysaccharide in *Acinetobacter* *baumannii*. Antimicrob Agents Chemother 55:3022–3024. doi:10.1128/AAC.01732-10. 21402838 PMC3101452

[29] Barb AW, Jiang L, Raetz CR, Zhou P 2007 Structure of the deacetylase LpxC bound to the antibiotic CHIR-090: time-dependent inhibition and specificity in ligand binding. Proc Natl Acad Sci U S A 104:18433–18438. doi:10.1073/pnas.0709412104. 18025458 PMC2141794

[30] McClerren AL, Endsley S, Bowman JL, Andersen NH, Guan Z, Rudolph J, Raetz CR 2005 A slow, tight-binding inhibitor of the zinc-dependent deacetylase LpxC of lipid A biosynthesis with antibiotic activity comparable to ciprofloxacin. Biochemistry 44:16574–16583. doi:10.1021/bi0518186. 16342948 PMC2742919

[31] Barb AW, Zhou P 2008 Mechanism and inhibition of LpxC: an essential zinc-dependent deacetylase of bacterial lipid A synthesis. Curr Pharm Biotechnol 9:9–15. doi:10.2174/138920108783497668. 18289052 PMC3022321

[32] Barb AW, McClerren AL, Snehelatha K, Reynolds CM, Zhou P, Raetz CR 2007 Inhibition of lipid A biosynthesis as the primary mechanism of CHIR-090 antibiotic activity in *Escherichia coli*. Biochemistry 46:3793–3802. doi:10.1021/bi6025165. 17335290 PMC2709454

[33] Moche M, Schneider G, Edwards P, Dehesh K, Lindqvist Y 1999 Structure of the complex between the antibiotic cerulenin and its target, beta-ketoacyl-acyl carrier protein synthase. J Biol Chem 274:6031–6034. doi:10.1074/jbc.274.10.6031. 10037680

[34] Miller JR, Dunham S, Mochalkin I, Banotai C, Bowman M, Buist S, Dunkle B, Hanna D, Harwood HJ, Huband MD, Karnovsky A, Kuhn M, Limberakis C, Liu JY, Mehrens S, Mueller WT, Narasimhan L, Ogden A, Ohren J, Prasad JV, Shelly JA, Skerlos L, Sulavik M, Thomas VH, VanderRoest S, Wang L, Wang Z, Whitton A, Zhu T, Stover CK 2009 A class of selective antibacterials derived from a protein kinase inhibitor pharmacophore. Proc Natl Acad Sci U S A 106:1737–1742. doi:10.1073/pnas.0811275106. 19164768 PMC2644107

[35] Bartling CM, Raetz CR 2008 Steady-state kinetics and mechanism of LpxD, the N-acyltransferase of lipid A biosynthesis. Biochemistry 47:5290–5302. doi:10.1021/bi800240r. 18422345 PMC2435086

[36] Zeng D, Zhao J, Chung HS, Guan Z, Raetz CR, Zhou P 2013 Mutants resistant to LpxC inhibitors by rebalancing cellular homeostasis. J Biol Chem 288:5475–5486. doi:10.1074/jbc.M112.447607. 23316051 PMC3581379

[37] Caughlan RE, Jones AK, Delucia AM, Woods AL, Xie L, Ma B, Barnes SW, Walker JR, Sprague ER, Yang X, Dean CR 2012 Mechanisms decreasing in vitro susceptibility to the LpxC inhibitor CHIR-090 in the gram-negative pathogen *Pseudomonas aeruginosa*. Antimicrob Agents Chemother 56:17–27. doi:10.1128/AAC.05417-11. 22024823 PMC3256010

[38] Clements JM, Coignard F, Johnson I, Chandler S, Palan S, Waller A, Wijkmans J, Hunter MG 2002 Antibacterial activities and characterization of novel inhibitors of LpxC. Antimicrob Agents Chemother 46:1793–1799. doi:10.1128/AAC.46.6.1793-1799.2002. 12019092 PMC127247

[39] Steeghs L, de Cock H, Evers E, Zomer B, Tommassen J, van der Ley P 2001 Outer membrane composition of a lipopolysaccharide-deficient *Neisseria meningitidis* mutant. EMBO J 20:6937–6945. doi:10.1093/emboj/20.24.6937. 11742971 PMC125796

[40] Yao J, Rock CO 2016 Bacterial fatty acid metabolism in modern antibiotic discovery. Biochim Biophys Acta doi:10.1016/j.bbalip.2016.09.014. PMC5364071 27668701

[41] Yao Z, Davis RM, Kishony R, Kahne D, Ruiz N 2012 Regulation of cell size in response to nutrient availability by fatty acid biosynthesis in *Escherichia coli*. Proc Natl Acad Sci U S A 109:E2561–E2568. doi:10.1073/pnas.1209742109. 22908292 PMC3458391

[42] Bushby SR 1975 Synergy of trimethoprim-sulfamethoxazole. Can Med Assoc J 112:63–66. 1093654 PMC1956440

[43] Thornton JA 2016 Splicing by overlap extension PCR to obtain hybrid DNA products. Methods Mol Biol 1373:43–49. doi:10.1007/7651_2014_182. 25646606

[44] Schneider CA, Rasband WS, Eliceiri KW 2012 NIH Image to ImageJ: 25 years of image analysis. Nat Methods 9:671–675. doi:10.1038/nmeth.2089. 22930834 PMC5554542

[45] Bojkovic J, Richie DL, Six DA, Rath CM, Sawyer WS, Hu Q, Dean CR 2015 Characterization of an *Acinetobacter* *baumannii* *lptD* deletion strain: permeability defects and response to inhibition of lipopolysaccharide and fatty acid biosynthesis. J Bacteriol 198:731–741. doi:10.1128/JB.00639-15. 26668262 PMC4751815

[46] MacLean B, Tomazela DM, Shulman N, Chambers M, Finney GL, Frewen B, Kern R, Tabb DL, Liebler DC, MacCoss MJ 2010 Skyline: an open source document editor for creating and analyzing targeted proteomics experiments. Bioinformatics 26:966–968. doi:10.1093/bioinformatics/btq054. 20147306 PMC2844992

