# Supplementary material for: LpxK Is Essential for Growth of Acinetobacter baumannii ATCC 19606: Relationship to Toxic Accumulation of Lipid A Pathway Intermediates
Source: mSphere. 2017 Jul 26;2(4):e00199-17. doi: 10.1128/mSphere.00199-17 (PMC5555675; doi:10.1128/mSphere.00199-17)
Supplement: TABLE S1 [file sph004172328st1.docx]

**Table S1**. Oligonucleotide sequences used in this study

| Primer designation | Primer Sequence (5' - 3') |
| --- | --- |
| KTT433 - US lpxK F | GTCCTGTTGTTCAAGTGGTAATGG |
| KTT434 - US lpxK-KanR R | GCAATTCCGGTTCGCTTGCTGTCACTTATTGGTCCTCAAAATTACGCTG |
| KTT435 - ptac-lpxK F | GGAAACAGAATTCGAGCTCGGTACATGTCTTTAGCCCAGCTGATCCAAAATG |
| KTT436 - DS lpxK R | GAAGCGTGCTGGAATGACGATATG |
| KTT238 - KanR-lacI F | GCCTTCTTGACGAGTTCTTCTGACAATTCGCGCTAACTTACATTAATTGC |
| KTT239 - ptac R | GTACCGAGCTCGAATTCTGTTTCC |
| KTT85 - KanR TOPO F | GACAGCAAGCGAACCGGAATTGC |
| KTT86 - KanR TOPO R | TCAGAAGAACTCGTCAAGAAGGC |
| cPCR lpxK F | AAGAGTCAGAACAAGAGCGTTTC |
| cPCR lpxK R | CACATAGGTCATCGAAACCTTCAAC |
